# Oligomerization triggered by foldon: a simple method to enhance the catalytic efficiency of lichenase and xylanase

**DOI:** 10.1186/s12896-017-0380-3

**Published:** 2017-07-03

**Authors:** Xinzhe Wang, Huihua Ge, Dandan Zhang, Shuyu Wu, Guangya Zhang

**Affiliations:** Fujian Provincial Key Laboratory of Biochemical Technology, Huaqiao University, Xiamen, Fujian 361021 China

**Keywords:** Foldon, Oligomerization, Non-chromatographic purification, Catalytic efficiency, Elastin-like polypeptides, Enzyme engineering

## Abstract

**Background:**

Effective and simple methods that lead to higher enzymatic efficiencies are highly sough. Here we proposed a foldon-triggered trimerization of the target enzymes with significantly improved catalytic performances by fusing a foldon domain at the C-terminus of the enzymes via elastin-like polypeptides (ELPs). The foldon domain comprises 27 residues and can forms trimers with high stability.

**Results:**

Lichenase and xylanase can hydrolyze lichenan and xylan to produce value added products and biofuels, and they have great potentials as biotechnological tools in various industrial applications. We took them as the examples and compared the kinetic parameters of the engineered trimeric enzymes to those of the monomeric and wild type ones. When compared with the monomeric ones, the catalytic efficiency (*k*_*cat*_ */K*_*m*_) of the trimeric lichenase and xylanase increased 4.2- and 3.9- fold. The catalytic constant (*k*_*cat*_) of the trimeric lichenase and xylanase increased 1.8- fold and 5.0- fold than their corresponding wild-type counterparts. Also, the specific activities of trimeric lichenase and xylanase increased by 149% and 94% than those of the monomeric ones. Besides, the recovery of the lichenase and xylanase activities increased by 12.4% and 6.1% during the purification process using ELPs as the non-chromatographic tag. The possible reason is the foldon domain can reduce the transition temperature of the ELPs.

**Conclusion:**

The trimeric lichenase and xylanase induced by foldon have advantages in the catalytic performances. Besides, they were easier to purify with increased purification fold and decreased the loss of activities compared to their corresponding monomeric ones. Trimerizing of the target enzymes triggered by the foldon domain could improve their activities and facilitate the purification, which represents a simple and effective enzyme-engineering tool. It should have exciting potentials both in industrial and laboratory scales.

**Electronic supplementary material:**

The online version of this article (doi:10.1186/s12896-017-0380-3) contains supplementary material, which is available to authorized users.

## Background

Enzymatic catalysis played a significant role in industry and laboratory, especially in enzymatic hydrolysis of lignocellulose to produce fuel-grade ethanol. It was an attractive opportunity for producing renewable and environmentally friendly biofuels [1]. Within this context, since the limited catalytic performance in the reaction process, many studies on the functional characteristics such as substrate affinity, high catalytic properties received extensive attention [2]. Presently, researchers have proposed some effective approaches to improve the catalytic performance of the enzymes. They included the site-directed mutagenesis and directed evolution, which has successfully produced enzymes with optimized features such as activity, thermal stability and substrate specificity etc. Sometimes, immobilization and chemical modification of the target enzymes could also achieve the goal [3].

The scale of improved enzymes produced by mutants and others methods existed is large. For example, Zhang [4] and coworkers revealed a series of xylanases mutants which displayed 35–45% decrease in *K*_*m*_ and 75–105% increase in *k*_*cat*_ and leading to an approximately 200% increase in catalytic efficiencies by directed evolution. And, mutating Asn to Asp at position 35 adjacent to Glu172 enhanced the catalytic activity of xylanase from *Bacillus circulans* [5]. Indeed, those approaches achieve great success for improving the enzyme activities to varying degrees. However, there are still several drawbacks as described below: (1). The process of directed evolution must be iterated until the desired change is reached, or until no further change is elicited iteratively for at least 2- rounds. It needs a straightforward and efficient high-throughput screening method [6, 7]; (2). Site-directed mutagens through rational approaches should base on structural analysis. It could not be achieved without the well-known of the relationship between crystal structure and functional amino acid residues [8]; (3). Chemical modification is that of covalent attachment of special groups of modifiers to the side-chain group of certain residues in the enzyme. This method is often in severe reaction conditions and may cause unexpected loss of enzymatic activity by alteration of the active conformation or essential residues in the active sites [9].

Our purpose is to develop a convenient and efficient enzyme engineering method to improve the catalytic activities based on trimerizing the target enzymes. Oligomerization is a general way for many proteins who self-associate into oligomers to gain functional advantages [10]. The subunit assembly induced by the domains such as collagen triple helices and the obligatory oligomers like COMP and foldon usually results in improving thermostability [11, 12]. Foldon was a small 27-residue (GYIPEAPRDGQAYVRKDGEWVLLSTFL) β-propeller like trimer consisting of monomeric β-hairpin segments, which was originally identified at the C-terminus of bacteriophage T4 fibritin [13]. By gene fusion, this domain may be artificially linked to target enzymes to change their properties. Thermodynamic stability of several engineered proteins such as short collagen fibers [14, 15], HIV1 envelope glycoprotein has been enhanced by means of attachment of the foldon domain [16, 17].

Here, we fused foldon at the C-terminus of the elastin-like polypeptides-lichenase (defined as monomeric lichenase) and elastin-like polypeptides-xylanase (defined as monomeric xylanase) to induce these monomeric enzymes forming trimeric enzymes, respectively. The insertion of the two domain was expected to make purifying the recombinant proteins more effective and trigger some improvement of the catalytic properties. We found the trimeric lichenase and xylanase showed superior kinetic parameters and improved catalytic activities over their corresponding monomeric and wild-type counterparts. Meanwhile, the trimeric lichenase and xylanase could improve the activity recovery during the process of non-chromatographic purification by elastin-like polypeptides (ELPs). ELPs are stimulus-responsive polymers consist of repeating pentapeptide (Gly-Xxx-Gly-Val-Pro), where X represents any amino acid except proline [18]. As a purification tag, ELPs were used to purify recombinant proteins and peptides without chromatography through undergoing an inverse transition cycling (ITC) within a narrow temperature range (2 ~ 3 °C) in aqueous solution [19]. The inverse phase transition can also be isothermally triggered by adding salt, which is a promising method both inexpensive and simple [20–22]. Also, the ELPs is in the random coil state when the target enzyme catalyzed the substrate, thus lessening the potentially unfavorable effects of the foldon domain on the active sites.

## Results

### Expression and purification of the recombinant lichenase and xylanase

**Fig. 1:**
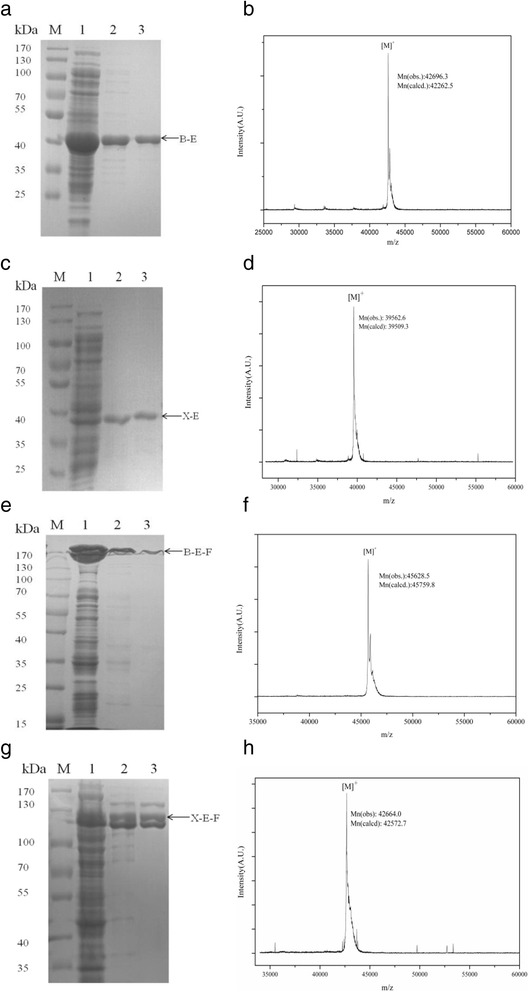
Purification and analyzed the target enzymes by SDS-PAGE and MALDI-TOF. Monomeric enzymes B-E **a**, **b** and X-E **c**, **d**; trimeric enzymes B-E-F **e**, **f** and X-E-F **g**, **h** Lane M: marker; lane1 the lysate; lane 2 supernatant of first-round ITC, lane 3 supernatant of second-round ITC

We successfully expressed the monomeric and trimeric genes, purified the target enzymes and evaluated their purities by gel electrophoresis. For the monomeric ones, SDS-PAGE yield one band of 43 kDa and 39 kDa, denoting the monomeric lichenase (B-E) and xylanase (X-E) with ELPs tag, respectively (Fig. 1a,c). Quantity calculating results demonstrated the monomeric lichenase and xylanase comprised about 98% and 99% of the total soluble proteins after purification. Besides, the precise molecular weights (MWs) of purified B-E and X-E were further determined by MALDI-TOF mass spectrometry (MS). They presented with MWs of 42,696.3 Da and 39,562.6 Da respectively (Fig. 1b,d), which matched their theoretical values at 42262.5 Da and 39,509.3 Da calculated by ProtParam (http://web.expasy.org/protparam/). As for the trimeric lichenase, SDS-PAGE yield one band of 137 kDa, it comprised about 95% of the total proteins (Fig. 1e). The precise MW of its monomeric constituent determined by MALDI-TOF MS was 45,628.5 Da, which was 2932.2 Da (about 3 kDa, the MW of the foldon) more than the MW of the B-E. It was exactly the MW of one subunit of foldon (Fig. 1f). When it comes to the trimeric xylanases, SDS-PAGE yield three close bands ranging from 110 kDa to 130 kDa, they comprised about 98% of the total proteins (Fig. 1g). The precise MW of it was 42,664.0 Da, which was also 3.1 kDa more than X-E, standing for one subunit of the foldon (Fig. 1h). The reason for three close bands in the SDS-PAGE might be the sample was not heated enough before loading on the gel and some of them refolded. However, we are sure they were all trimeric xylanases. Because we only detected one molecule weight (42,664.0 Da) by the MALDI-TOF MS, which was exactly the MW of the monomer of the X-E-F, indicating there were no covalent bonds between monomers.

**Fig. 2:**
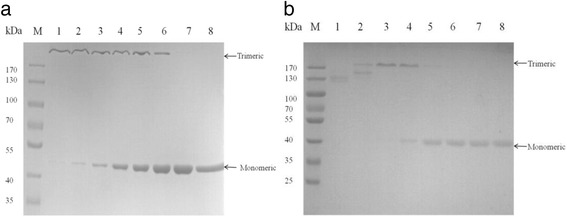
The influences of temperature on the oligomeric state of lichenase and xylanase. The samples of trimeric lichenase **a** xylanase **b** were heated for 5 min at the designated temperatures prior to loading on the gel. The temperature was set to 25 °C (lane 1), 40 °C (lane 2), 50 °C (lane 3), 55 °C (lane 4), 60 °C (lane 5), 65 °C (lane 6), 70 °C (lane 7) and 100 °C (lane 8), respectively

To illustrate the thermal stability of the trimeric enzymes, we pretreated them with different temperatures (ranging from 25 °C to 100 °C) in loading buffer as reported [23] and then evaluated by gel electrophoresis. As Fig. 2a shows, when we pretreated the trimeric lichenase at the temperature of 25 °C, SDS-PAGE yielded one band of 137 kDa. It was about 3 times of the MWs of the monomer (46 kDa), indicating the trimeric lichenase remains folded. However, as the temperature changes from 40 °C to 70 °C, the trimeric lichenase began to unfolded partially, and thus the SDS-PAGE yielded two bands. Of them, one was the trimeric one and the other was the monomeric one with the MWs about 46 kDa. When the temperature is 70 °C or above, the SDS-PAGE yielded one band of 46 kDa, indicating the trimeric lichenase unfolded to the monomers. Besides, we also observed the unfolding monomers refolded into trimer after a rapid cooling in spite of withstanding the high temperature of 100 °C. Similar trends also existed in the trimeric xylanase as shown in Fig. 2b. However, there was one thing needed to point out. The SDS-PAGE yields three close bands when the trimeric xylanase was pretreated under the temperature of 40 °C, it yields two close bands under the temperature ranging from 50 °C to 65 °C, and it finally yields 1 bands when we pretreated the trimeric xylanase above 70 °C. These results proved the three bands in lane 1 and 2 were both the trimeric xylanase, which agreed with the results mentioned above.

We also compared the specific activity, purification fold and the activity recovery of the trimeric and monomeric enzymes during the ITC purification process and listed the results in Table 1. From it, we can see the trimeric enzymes are easier to purify with increased purification folds and activity recovery. For example, the purification fold of B-E-F and X-E-F was 7.4 and 12.5, which is 1.7 and 2.1 times of the monomeric ones. Meanwhile, the activity recovery increased by 12.5% and 6.2%, respectively. As shown in Table 1, the specific activity of the trimeric lichenase and xylanase was 88.2 ± 3.5 and 516.0 ± 10.3 U/mg, which enhanced 149% and 94% than their corresponding monomer ones. These results demonstrated the foldon domain was beneficial to the purification of the target enzyme when it fused at the C-terminus of the ELPs tag. Considering the ELPs system can facilitate purifying enzymes (proteins) from the cell lysate and eliminates the need for expensive chromatography, our method may have great potentials in the recombinant enzyme (protein) bio-separation technology ranging from laboratory to manufacturing scales applications [24].

**Table 1:** Purification performance of the monomeric and trimeric enzymes

ITC purification		Specific activity(U/mg)	Purification fold	Recovery of activity (%)
B-E-F	Crude	11.9 ± 0.5	7.4	68.7 ± 2.2
	Purified	88.2 ± 3.5		
B-E	Crude	8.1 ± 0.8	4.4	56.2 ± 1.4
	Purified	35.4 ± 0.9		
X-E-F	Crude	41.4 ± 0.1	12.5	66.3 ± 1.4
	Purified	516.0 ± 10.3		
X-E	Crude	45.4 ± 0.1	5.8	60.2 ± 2.8
	purified	265.2 ± 3.9		

### Optimal temperature and pH of the trimeric and monomeric enzymes

**Fig. 3:**
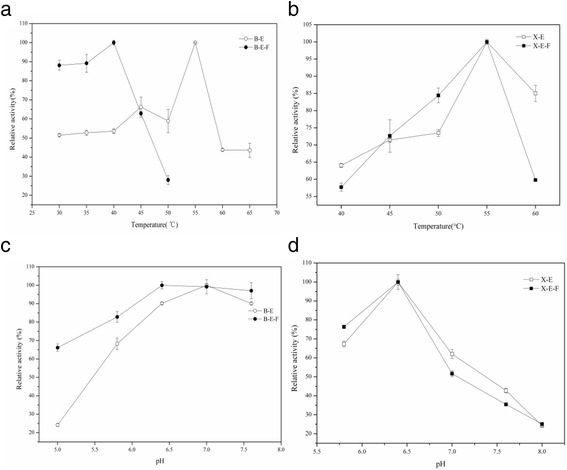
The influences of temperature and pH on the relative activity of lichenase and xylanase. The influences of temperature on recombinant monomeric and trimeric lichenase **a** and xylanase **b** and the influences of pH on the relative activity of recombinant monomeric and trimeric lichenase **c** and xylanase **d** The error *bars* indicate the standard error of the mean (SEM)

To evaluate the optimal temperature and pH profiles, we assayed the activities of the recombinant enzymes at various temperatures ranging from 30 °C to 65 °C, or pH ranging from 5.0 to 8.0. The relative activity was calculated by comparing to the highest enzyme activity (defined as 100%) to assure the optimal condition. For B-E-F and B-E, they have consistent optimal pH at 6.4–7.0 but the different optimal temperature of 40 °C and 55 °C, respectively (Fig. 3a,c), while the optimum pH and temperature of the wild type were 7.0 and 50 °C. For X-E-F and X-E, they show the same pH and temperature optimum of 6.4 and 55 °C (Fig. 3b,d), respectively. They were very similar with those of the wild-type xylanases, which had the optimal pH and temperature of 7.0 and 50 °C, respectively. For the thermostability, we assayed the residual activity of lichenase after incubated at the optimum condition for 4 h. The trimeric one remained about 65% of the relative activity, while the monomeric only 40%. In addition, the relative activity of monomeric xylanases decreased faster than the trimeric ones. Notably, the acid resistance of B-E-F was sharply improved. It remained 65% of the relative activity at pH 5.0, while B-E only remained 20%. Similarly, as previously reported, better pH resistance of GFP was obtained by formation of tunable dimerization, suggesting that protein conformations, in general, may be used to alter other protein properties [25].

### Trimeric enzymes with improved kinetic parameters

**Fig. 4:**
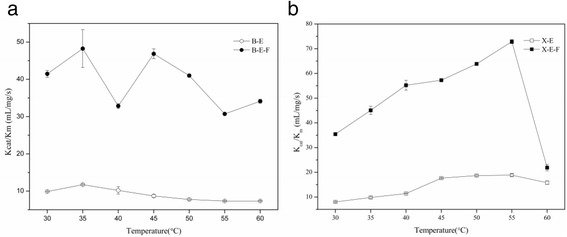
The *k*_*cat*_ */K*_*m*_ of lichenase and xylanase at different temperatures. *K*_*cat*_ */K*_*m*_ of monomeric and trimeric lichenase **a** and xylanase **b** at various temperature from 30 to 60 °C, samples were performed at 5 °C intervals. The error bars indicate the standard error of the mean (SEM)

We measured the kinetic parameters of the recombinant enzymes at their own optimal temperature and pH (shown in Table 2.) and compared them with the wild type ones previously reported [26]. As our sequence came from it and we synthesized the gene coding the identical lichenase and xylanase with the reference. The *K*_*m*_ values of the B-E-F and X-E-F for glucan and birchwood xylan are 61.1 ± 2.8 mmol/L and 79.5 ± 4.0 mmol/L, which decreases 1.4- and 1.7- fold respectively when compared with their corresponding monomeric ones. This indicates the trimeric enzymes have the better affinity to their specific substrates than the monomeric ones. Besides, the catalytic constant (*k*_*cat*_) of B-E-F is 361.2 ± 24.0 s^−1^, which increases 3.0- and 1.8- fold according to their monomeric and wild-type lichenase. The *k*_*cat*_ of X-E-F is 870.1 ± 8.9 s^−1^, which increases 2.3- and 5.0- fold according to their monomeric and wild-type xylanase, respectively. As mentioned above, the foldon domain in the trimeric lichenase and xylanase were partially unfolded at the temperature ranging from 45 °C to 60 °C. To compare the catalytic properties changed by oligomerization, we measured the catalytic efficiency (*k*_*cat*_ */K*_*m*_) over temperatures ranging from 30 to 60 °C. As Fig. 4 showed, the trimeric enzymes displayed distinguished catalytic efficiency (*k*_*cat*_ */K*_*m*_) than those of their corresponding monomeric ones in the measured temperature range. What needs to point out is the *k*_*cat*_ of the monomeric xylanase is higher (2.2 times) than the wild-type enzyme. Similar reports also existed in other researcher’s results. For example, Yang and coworkers fused an oligopeptide to the N-terminus of an alkaline- amylase, the specific activity and catalytic constant (*k*_*cat*_) of AmyK-p1 increased by 4.1- and 3.5-fold, respectively. They thought the main reason for the improved catalytic efficiency and the specific activity is the greater flexibility around the active site induced by the oligopeptide [27, 28]. Here, the catalytic efficiency (*k*_*cat*_ */K*_*m*_) of the monomeric xylanases is lower than the wild type although the values are quite similar, this is due to the increased *K*_*m*_ of the monomeric xylanases. For lichenase, the catalytic constant (*k*_*cat*_) of the wild-type is higher than the monomeric although the values are quite similar. However, the catalytic efficiency of the monomeric lichenase is lower than the wild-type, this is also because of the increased *K*_*m*_ of the monomeric lichenase. The decrease of the substrate affinity of the engineered enzymes might relate to the fact the designed repeated pentapeptides were larger than the frequently used tag like histidine. It may have potential steric hindrance when the target enzyme binds the substrate.

**Table 2:** Kinetic parameter of trimeric, monomeric and wild-type enzymes

		*K*_*cat*_ (s^−1^)	*K*_*m*_ (mmol/L)	*K*_*cat*_ */K*_*m*_ (L/mmol/s)
Lichenase	Wild type	197.2 ± 8.5	18.8 ± 1.8	10.5 ± 0.5
	Trimeric	361.2 ± 24.0	61.1 ± 2.8	5.9 ± 0.4
	Monomeric	119.7 ± 8.8	85.6 ± 4.0	1.4 ± 0.1
Xylanase	Wild type	173.7 ± 8.3	51.8 ± 4.6	3.4 ± 0.2
	Trimeric	870.1 ± 8.9	79.5 ± 4.0	10.9 ± 0.4
	Monomeric	379.8 ± 12.1	134.2 ± 14.7	2.8 ± 0.1

## Discussions

Enzyme engineering has become a common strategy to optimize the catalytic and biophysical properties of enzymes, and the improved performance makes biocatalysts attractive in both the laboratory and industrial processes. Directed evolution and site-directed mutagenesis techniques have been used with great success for diversifying gene sequences and optimizing enzyme phenotypes. Most of the engineered enzymes are those with improved biophysical properties, such as improved stability to high temperature or harsh pH, few of them with increased catalytic activities. For example, Zhang and coworkers conducted site-directed mutagenesis of β-1, 4-endoglucanase based on a rational design and obtained increased activity of 77% -87%. The *V*_*max*_ and *K*_*m*_ of the triple-sites mutant were 4.23 ± 0.15 μmol mg^−1^ min^−1^ and 1.97 ± 0.05 mM, respectively, about 2 times higher than those of the initial enzyme [29]. Chemical modification of the target enzyme is another effective way to increase the catalytic activities of some thermophilic enzymes. For instance, Lys modification of α-amylase from *Bacillus licheniformis* led to a 15-fold increase in activity of BLA at 15 °C with a 3-fold increase in *k*_*cat*_ */K*_*m*_ at 37 °C [30]. Besides, Lin et al. obtained a mutant Q51H of glutamate decarboxylase with higher *k*_*cat*_ and *k*_*cat*_ */K*_*m*_ of 47.67 ± 3.18 s^−1^ and 2.05 ± 0.09 s^−1^· (mmol·L^−1^)^−1^ compared to 18.06 ± 1.70 s^−1^ and 0.80 ± 0.06 s^−1^·(mmol· L^−1^)^−1^ of the parental, respectively [31]. According to the previous results, our method is comparable with those conducted with enzyme mutagenesis or modification. However, our method is in mild condition, simple to operate and do not need to screen target enzymes from the mutant libraries. It should be a simple enzyme engineering method or at least be an effective complement to the existing methods.

Many researchers have illustrated subunit oligomerization motifs play an important role in protein function. Such as the parallel five-stranded coiled coil COMP, the obligatory dimerization POZ, the foldon domain and so on [11, 12]. For example, the thermal hysteresis activity of the antifreeze proteins (AFPs) was increased significantly, reaching a thermal hysteresis of >1.6 °C at the concentration of <1 mM [32]. Another example is the melting temperature (Tm) of a collagen-like peptide increased to around 75 °C from 24 °C when attaching a foldon domain to the C-terminus [33].

As the results displayed in this work, the trimeric lichenase and xylanase showed distinguished substrate affinity and superior catalytic efficiency likely accelerated owing to the high intrinsic concentration and diminished enthalpic interactions imposed by the trimeric foldon clamp [15]. Zanphorlin and coworkers have just recently shown such increased activity and *k*_*cat*_. In their work, they showed that a tetramer leading to a 10-fold higher activity with higher *k*_*cat*_ compared to the disassembled monomers of the psychrophilic β-glucosidases [34]. However, the tetramer is the natural biological active unit of the β –glucosidase. While the monomers, which was just a small fraction of the predominant population of tetramers, were obtained during the size-exclusion chromatography. The monomer is about ten-fold less active than the tetramer, suggesting the quaternary structure is crucial for the proper function of this enzyme. Recently, Eshaghi and coworkers obtained dimeric fluorescent proteins based on a single-domain antibody, which led 7- to 8-fold increased pH resistance, and 3- fold higher brightness in vitro. The structural basis for improved brightness and acid resistance relies on stabilization of unfavorable protein conformations [25]. Besides, Yang and coworkers obtained trimeric nitrilase active inclusion bodies by fusing an amphipathic self-assembly peptide 18A at the C-terminus of nitrilase, which has higher specific activities and thermostability than the native nitrilase [35]. Our method is a little bit similar to their work and has a comparable improvement of activity but with different oligomerization trigger domain. The improved performance of the trimeric enzymes is likely accelerated owing to the increase in the local concentration of the enzymes. And it also relied on stabilizing the protein conformations triggered by the obligatory domain foldon, which accelerated the contact of active site and substrate [36]. These results are very encouraging, as the foldon-guided trimerization of the target enzymes will lead to improving their catalytic performances effectively and simply.

Thus, our results are the first report about increasing the activities of lichenase and xylanase by fusing a foldon domain at the C-terminus to trigger their trimerization. It should also be applicable to other enzymes by proper design. Thanks to the cheap and efficient gene synthesis technology, our methods without chemical modification and mutations was effective, simple and powerful. Perhaps, further research should pay more attention to understand why and how the oligomerization domains improve the functional properties of target enzymes. Besides, the relationship between the oligomeric states and the activity of the target enzymes is also an interesting area of concern and need more investigation.

It is well known that many factors could affect the thermal behaviors of ELPs, and the physical properties of the protein fused with ELPs was one of them. The foldon domain was expected to reduce the transition temperature of the ELPs because of increased chain length, molecular weight, intrinsic concentration and enhanced hydrophobic interactions [37]. We observed the transition temperatures of B-E-F and X-E-F decreased by 8 °C and 11 °C than those of B-E and X-E, respectively. This feature is beneficial for achieving effective purification at low salinity concentrations and temperature for the ELPs containing oligomerization domain.

The increased hydrophobic character, as well as increased concentration of ELPs, promoted chain folding and aggregation, and thus oligomerization of the target enzymes facilitated functional advantages such as superior catalysis properties, increased recovery of activity and specific activity. However, a latent problem is whether oligomerization of protein could be potentially adopted as a general strategy to obtain multiple functions in various enzymes. As the foldon domain in samples was unfolded when heated to 70 °C or above, it may not suitable for the thermophilic enzyme who have the optimal temperature above the unfolding temperature of foldon (70 °C). Therefore, enzymes with proper optimum temperature will be studied to verify the properties and mechanism changed by oligomerization domain in the future. Besides, introducing the disulfide bond in the foldon domain may improve its thermostability, thus the foldon-triggered oligomerization methods we proposed here may be useful for improving the catalytic performances of the thermophilic enzymes.

## Conclusion

Our results presented here highlight two important properties of the foldon-triggered trimerization of the lichenase and xylanase. First, the trimeric enzymes induced by foldon have advantages in catalytic performances such as improved activity, catalytic efficiency and substrate affinity over the monomeric ones. Second, the foldon domain could increase the purification fold and decrease the loss of activity during the process of ITC purification. The foldon domain should be an effective tool to improve the catalysis performance and purification efficiency of the target enzymes, and it will have great potentials in enzyme engineering and purification.

## Methods

### Plasmid construction

The complete genetic sequence of lichenase (bglS, Gene ID: 937,470) and xylanase (xynA, Gene ID: 939,861) came from references [26]. They were synthesized and sequenced by Sangon Biotech Co., Ltd. (Shanghai, China) and then cloned into pET 22(b+) vector. ELPs_40_ (VPGVG) was preserved in our lab and fused with the C-terminal of the target enzymes. Meanwhile, the gene encoding the monomeric ELPs-fused enzymes was ligated between the *Ned*I and *Hind*III restriction sites of pET 22b(+), respectively (denoted as B-E and X-E). For comparison, we also constructed a DNA fragment that encodes the trimeric enzymes. The foldon gene directly fused with the C-terminal of ELPs, which was fused at the C-terminal of the enzymes using *Ned*I and *Hind*III as the restriction sites as well (denoted as B-E-F and X-E-F). The gene encoding the foldon domain was synthesized by Sangon Biotech Co., Ltd. (Shanghai, China) by means of overlapping PCR amplification. The recombinant plasmids encoding the lichenase and xylanase from *Bacillus subtilis* 168 were transformed into *E.coli* BL21. Recombinant plasmids of xylanase were shown in Additional file 1: Figure S1 and Additional file 2: Figure S2.

### Protein expression, extraction, and purification

Plasmids carrying the monomeric and trimeric enzymes were transformed and overexpressed in the *E. coli* strain BL21(DE3). Single colonies were incubated overnight (~12 h) at 37 °C in fresh Luria Bertani (LB) supplemented with 100 μg/ml ampicillin. And then inoculated into terrific broth (TB) medium for a continued incubation at 37 °C till optical density at 600 nm of the culture reached between 0.4 and 0.6. The culture was induced for 20 h at 20 °C with 0.5 mmol/L isopropyl-β-thiogalactopyranoside (IPTG). Cell pellets were collected by centrifugation at 4 °C (8000 g, 10 min), resuspended in cold 0.1 mol/L phosphate-buffered saline (PBS, pH 6.4) and lysed by ultrasonic disruption at 4 °C. We harvested the crude enzymes by centrifugation at 4 °C (8000 g, 10 min) to remove insoluble cell debris and obtained supernatant. The fusion proteins were purified by ITC, as described previously [17]. Briefly, the inverse phase transition of the ELPs-fused enzymes was triggered by adding crystalline NaCl (2.5 mol/L) and further incubated at 40 °C for 20 min. The aggregations were separated from the lysate by centrifugation at 40 °C (12,000 g, 10 min) immediately. After discarding the supernatant, the pellets containing the fusion enzymes were resuspended in cold PBS, and another centrifugation at 4 °C (12,000 g, 10 min) was performed to remove the insoluble portion and collect the supernatant from the spin. Two steps constituted one round of ITC and the aggregation and resolubilization process were repeated twice [38]. The proteins purity was firstly determined with the 12% sodium dodecyl sulfate polyacrylamide gel electrophoresis (SDS-PAGE) with commassie brilliant blue staining, and then the UltrafleXtreme MALDI TOF mass spectrometer (Bruker Daltonics Inc., MA, USA) with sinapinic acid as the matrix. We removed the salts using the ultrafiltration centrifuge tube (Millpore). The protein concentration was measured by Coomassie brilliant blue method using bovine serum albumin serum (BSA) as a standard.

### Enzyme activity assays

The activities of the lichenase and xylanase were determined by measuring the release of reducing sugars from the substrates by the dinitrosalicylic acid (DNS) procedure and monitored with the OD value at the wavelength of 540 nm [39]. The standard process was conducted at their respective optimum temperature for 10 min in 0.1 mol/L PBS, using 1% (*w*/*v*) lichenan (Megazyme, Wicklow, Ireland) or birchwood xylan (Sigma) as substrate respectively. One activity unit (U) was defined as the amount of enzyme releasing 1 mol of reducing sugar per minute under the assay condition. Purified lichenases and xylanases were used throughout the assays and all the assays were performed in triplicate.

To determine the optimal pH reaction condition, the enzymatic reaction was conducted at different pH ranging from 5.0 to 8.0 in PBS. The optimal temperature was determined by incubating the reaction mixture for 10 min at pH 6.4 at a temperature gradient ranging from 30 to 65 °C.

### Enzyme kinetic parameters determination

We calculated the kinetic parameters by using substrate concentrations prepared at different concentrations (0, 1.1, 1.25, 1.43, 1.67, 2, 2.5, 3.33, 5, 10 g L^−1^) in PBS with pH 6.4. A given volume of lichenase or xylanase was added to their corresponding substrate solutions and incubated for 10 min, respectively. All the enzymatic reactions were conducted at the optimal temperature and pH. The substrate saturation curves were fitted into Michaelis–Menten kinetics and the *K*_*m*_ value and *V*_*max*_ of lichenase and xylanase were calculated using Lineweaver–Burk plot.

## Additional files

Additional file 1: Figure S1. The profiles of plasmid lichenase. The profiles of plasmid monomeric lichenase **a**, the gene was cloned between *Ned*I and *Hind*III digestion sites in pET 22b(+); plasmid trimeric lichenase **b**, foldon was directly fused with the *Hind*III digestion sites in pET 22b(+). (docx 3190 KB)

Additional file 2: Figure S2. The profiles of plasmid xylanase. The profiles of plasmid monomeric xylanase **a**, the gene was cloned between *Ned*I and *Hind*III digestion sites in pET 22b(+); plasmid trimeric xylanase **b**, foldon was directly fused with the *Hind*III digestion sites in pET 22b(+). (docx 3750 kb)

## Abbreviations

BSA: Bovine serum albumin
DNS: 3, 5-dinitrosalicylic acid
ELPs: Elastin-like polypeptides
IPTG: Isopropyl-β-thiogalactopyranoside
ITC: Inverse transition cycling
LB: Luria-Bertani
MALDI-TOF: Matrix-assisted laser desorption ionization time of flight
MS: Mass spectrometer
MW: Molecular weight
PBS: Phosphate-buffered saline
SDS-PAGE: Sodium dodecyl sulfate polyacrylamide gel electrophoresis
TB: Terrific broth
Tm: Melting temperature
Tt: Transition temperature
