# Supplementary figures and images for: Oligomerization triggered by foldon: a simple method to enhance the catalytic efficiency of lichenase and xylanase
Source: BMC Biotechnol. 2017 Jul 3;17:57. doi: 10.1186/s12896-017-0380-3 (PMC5496177; doi:10.1186/s12896-017-0380-3)

a.

b.


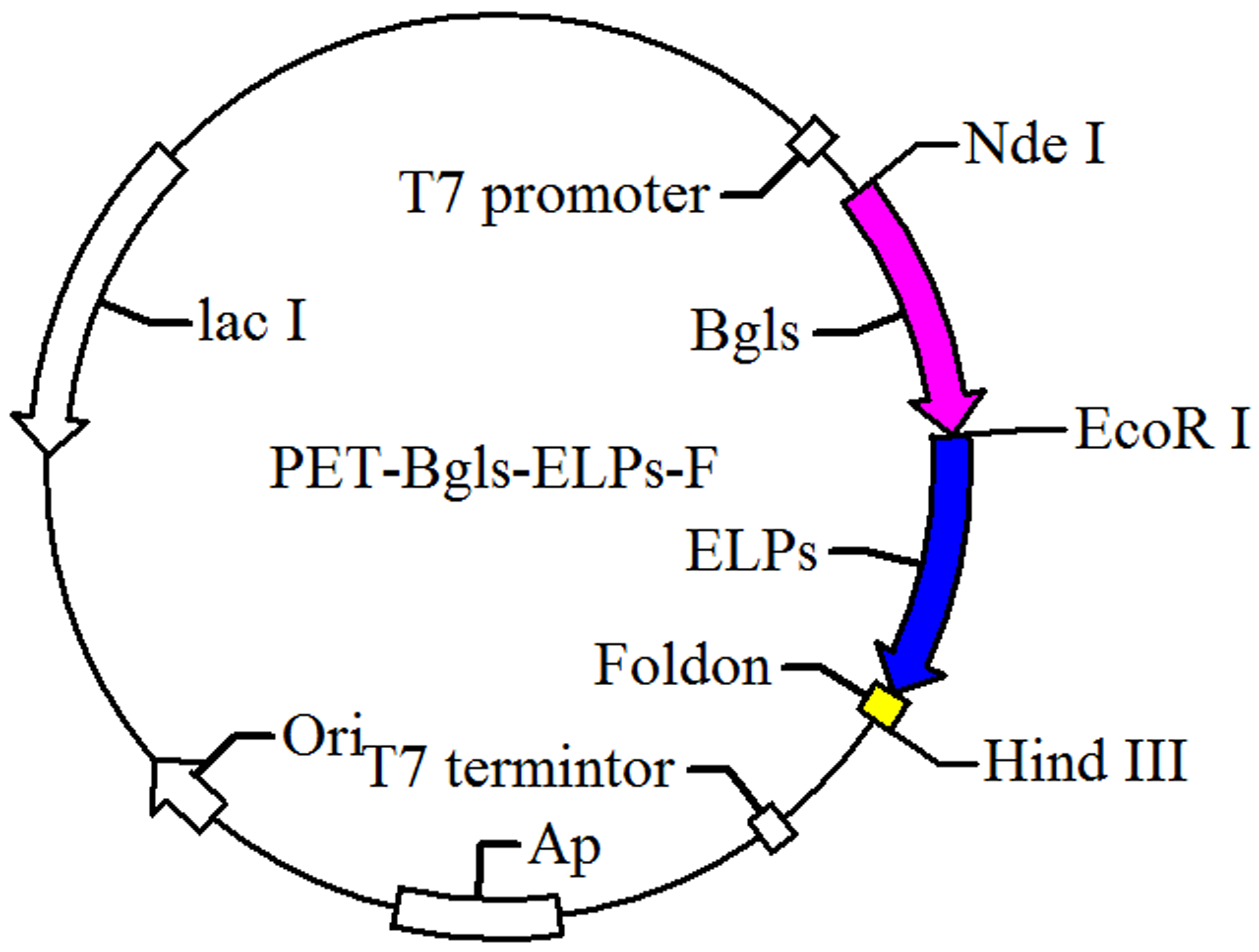

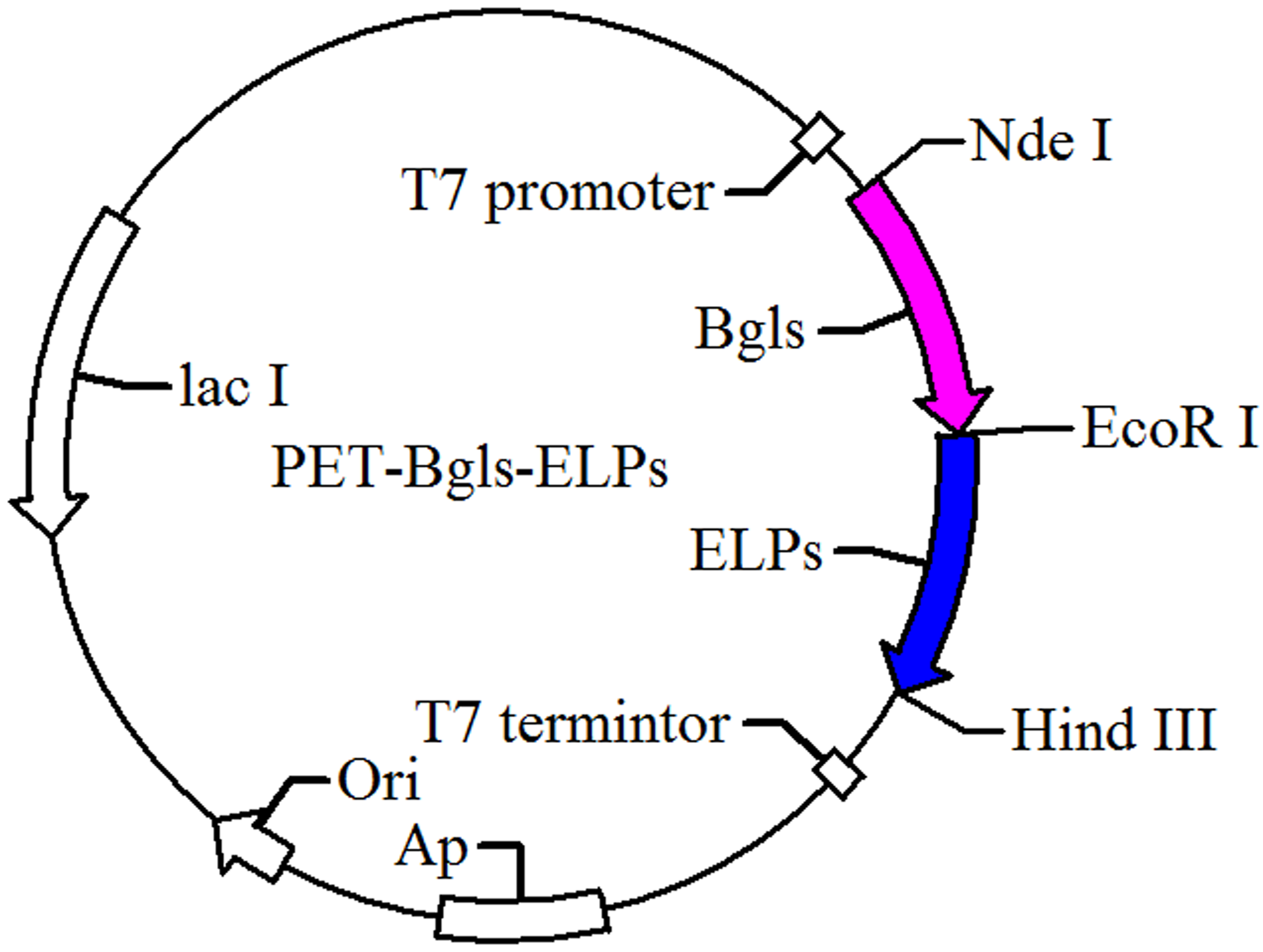


Figure. S1

Supplement: Supplementary file 1 — The profiles of plasmid lichenase. The profiles of plasmid monomeric lichenase a, the gene was cloned between NedI and HindIII digestion sites in pET 22b(+); plasmid trimeric lichenase b, foldon was directly fused with the HindIII digestion sites in pET 22b(+). (docx 3190 KB) [file 12896_2017_380_MOESM1_ESM.docx]

b.

a.


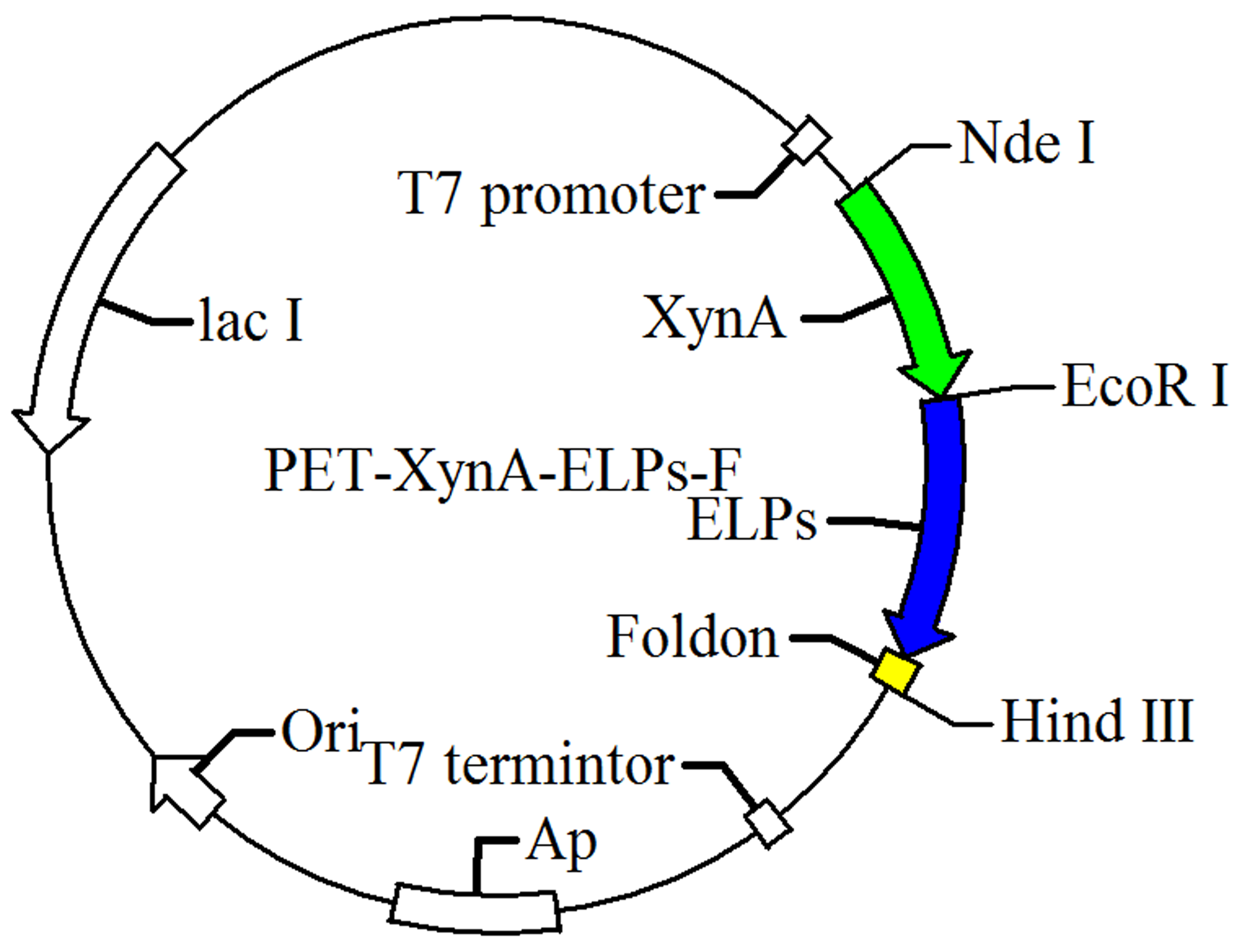

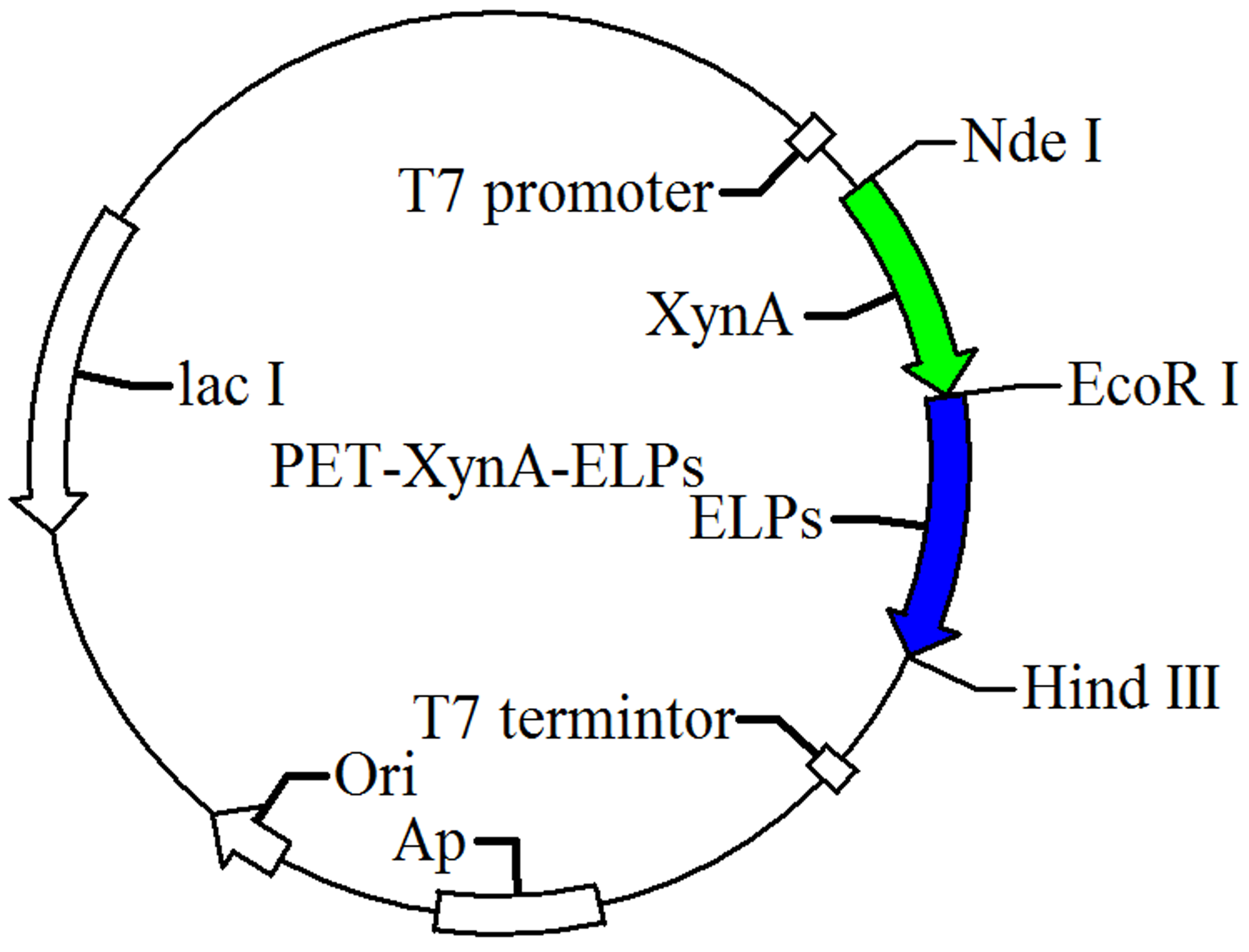


Figure. S2

Supplement: Supplementary file 2 — The profiles of plasmid xylanase. The profiles of plasmid monomeric xylanase a, the gene was cloned between NedI and HindIII digestion sites in pET 22b(+); plasmid trimeric xylanase b, foldon was directly fused with the HindIII digestion sites in pET 22b(+). (docx 3750 kb) [file 12896_2017_380_MOESM2_ESM.docx]
